# Safety of Ertugliflozin in Patients with Type 2 Diabetes Mellitus Inadequately Controlled with Conventional Therapy at Different Periods: A Meta-Analysis of Randomized Controlled Trials

**DOI:** 10.1155/2020/9704659

**Published:** 2020-12-14

**Authors:** Jing Huang, Shuyuan Xiong, Shenglan Ding, Qingfeng Cheng, Zhiping Liu

**Affiliations:** Department of Endocrinology, The First Affiliated Hospital of Chongqing Medical University, Chongqing 400016, China

## Abstract

**Aims:**

To assess the safety of ertugliflozin in patients with type 2 diabetes mellitus (T2DM) inadequately controlled with conventional therapy at different periods.

**Methods:**

We searched PubMed, Embase, and The Cochrane Library from inception to September 23, 2020. A total of six studies involving 4120 patients were included.

**Results:**

Compared with the control group, 15 mg and 5 mg of ertugliflozin were associated with higher risks of genital mycotic infections (GMIs) at 26 weeks (*p* < 0.0001 and *p* < 0.0001, respectively), 52 weeks (*p* < 0.00001 and *p* < 0.0001, respectively), and 104 weeks (*p* < 0.00001 and *p* < 0.0001, respectively). Moreover, females had a higher risk of GMIs than males in the 15 mg group at 26 weeks (*p* = 0.0008), 52 weeks (*p* < 0.0001), and 104 weeks (*p* = 0.02). At 104 weeks, 15 mg and 5 mg of ertugliflozin showed beneficial effects on symptomatic hypoglycemia (*p* < 0.00001 and *p* = 0.004, respectively) compared with the effects observed in the control group. Compared with the control group, 15 mg and 5 mg of ertugliflozin were associated with higher risks of drug-related adverse events at 26 weeks (*p* = 0.002 and *p* = 0.002, respectively); 15 mg of ertugliflozin was associated with a higher risk of discontinuation related to adverse events at 104 weeks (*p* = 0.03). No significant differences were found in the remaining safety outcomes.

**Conclusion:**

This meta-analysis of randomized controlled trials indicates that ertugliflozin is tolerated by T2DM, but the risk of GMIs is noteworthy, especially among females in the high-dose group.

## 1. Introduction

In diabetes mellitus, the global estimates of its prevalence are increasing each year, with T2DM accounting for approximately 90% of cases [1]. Although many antihyperglycemic agents have already been available for the treatment of T2DM, their glucose-lowering effects in the context of long-term glycemic control are not satisfactory [2]. There is an urgent need for more effective agents with fewer adverse effects to lower blood glucose.

Sodium glucose cotransporter 2 (SGLT2) inhibitors are a novel kind of antihyperglycemic agent, approved by the US Food and Drug Administration (FDA) to use with diet and exercise to lower blood glucose in adults with type 2 diabetes. Medicines in the SGLT2 inhibitor category were first approved in 2013, including canagliflozin, dapagliflozin, empagliflozin, and ertugliflozin [3]. Ertugliflozin is the fourth SGLT2 inhibitor approved in the United States [4]. By decreasing the renal glucose threshold, therefore increasing urinary glucose excretion, the pharmacological inhibition of SGLT2 cotransporters reduces hyperglycemia, offering an effective way to treat T2DM patients [5]. Ertugliflozin as monotherapy [6] or in combination [7] with other antihyperglycemic agents has been associated with improvements in glycemic control, body weight, and blood pressure.

However, in 2018, the FDA issued a warning that SGLT2 inhibitors reported cases of severe genital infections [3]. Although ertugliflozin has a good hypoglycemic effect, genital infection is an adverse event that deserves attention. In recent years, several studies have evaluated the efficacy and safety of ertugliflozin in T2DM. Ertugliflozin exhibited advantages for T2DM but at the same time increased risks for adverse events, such as GMIs [8, 9]. However, there is currently a lack of meta-analysis only for the safety of ertugliflozin, and the safety of ertugliflozin as a monotherapy at different periods with different doses was unclear and needs to be investigated.

Thus, we performed a meta-analysis of randomized controlled trials to assess the safety of ertugliflozin monotherapy at doses of 15 mg and 5 mg on GMIs, urinary tract infections (UTIs), drug-related adverse events, drug-related serious adverse events, discontinuation related to adverse events, deaths, symptomatic hypoglycemia, and hypovolemia at 26, 52, and 104 weeks. We also compared the effects of 15 mg ertugliflozin with a 5 mg dose on safety outcomes in each period. The registration number is CRD42020211388.

## 2. Materials and Methods

This study was conducted according to the Cochrane Collaboration and the PRISMA (Preferred Reporting Items for Systematic Reviews and Meta-Analyses) statement [10, 11].

### 2.1. Eligibility Criteria

Studies satisfying the following criteria were included:

1. *Population*: patients were diagnosed as T2DM according to American Diabetes Association guidelines and were ≥18 years old and had glycated hemoglobin that was inadequately controlled with conventional therapy (metformin or diet and exercise).
2. *Intervention*: monotherapy ertugliflozin at doses of 15 mg and 5 mg with or without a background of metformin; the treatment period was 26, 52, or 104 weeks.
3. *Comparison*: other hypoglycemic agents or placebo.
4. *Outcome*: GMIs, UTIs, drug-related adverse events, drug-related serious adverse events, discontinuation-related adverse events, deaths, symptomatic hypoglycemia, and hypovolemia.
5. *Study design*: only randomized controlled trials (RCTs) were included.
6. *Language restrictions*: only studies published in English.

The exclusion criteria were as follows: patients with type 1 diabetes mellitus, a history of ketoacidosis, an estimated glomerular filtration rate (eGFR) < 60 ml/min/1.73 m^2^, or a history of cardiovascular events within 3 months of screening.

### 2.2. Search Strategy

A systematic literature search about RCTs of ertugliflozin was conducted by two investigators (HJ and XSY) to identify relevant studies on PubMed, Embase, and The Cochrane Library from inception to September 23, 2020. The language was restricted to English. We also searched ClinicalTrials.gov and reviewed the references of the included articles to identify additional studies. The search terms included “diabet^∗^” and “ertugliflozin.” After eliminating the duplicates, the two investigators screened the titles and abstracts independently. Then, they performed a full-text evaluation. Meanwhile, discrepancies were referred to a third investigator and resolved through discussion (DSL).

### 2.3. Data Extraction

Data was extracted using a tailored form, including the following: the first author, publication year, NCT number, HbA1c% (baseline), the number of patients, intervention, and safety outcomes (primary: GMIs and UTIs; secondary: drug-related adverse events, drug-related serious adverse events, discontinuation related to adverse events, deaths, symptomatic hypoglycemia, and hypovolemia). The data selection procedure was performed independently by two investigators (HJ and XSY), and discrepancies were resolved through discussion among the three investigators (HJ, XSY, and DSL).

### 2.4. Risk of Bias and Strength of Evidence

The Cochrane Risk of Bias Tool was used to assess the risk of bias for included studies [12]. Each included study was assessed as “high,” “low,” or “unclear” risk of bias based on the following seven domains: random sequence generation, allocation concealment, blinding of participants and personnel, blinding of outcome assessment, incomplete outcome data, selective reporting, and other bias. Furthermore, the strength of evidence for each outcome (ertugliflozin group vs. control group) was judged as high, moderate, low, or very low according to the Grading of Recommendations, Assessment, Development and Evaluations (GRADE) system [13], and each study's quality was decreased based on risk of bias, inconsistency, indirectness, imprecision, and publication bias (GRADEpro GDT, https://gdt.gradepro.org/). Two investigators (HJ and XSY) reviewed and classified the RCTs independently. Differing opinions were resolved through discussions with a third investigator (DSL).

### 2.5. Statistical Analysis

Review Manager 5.3 was used for meta-analysis. Since all of the extracted data were dichotomous, risk ratio (RR) and 95% confidence interval (CI) were calculated to estimate effect size for dichotomous variables. *p* values <0.05 were deemed statistically significant. The backgrounds of the patients and control groups of included studies were not homogeneous. Taking into account the heterogeneity between studies, a random effects model was used to aggregate data to promote the generality of the results. A sensitivity analysis was performed by excluding studies one by one. Due to the small number of included studies, subgroup analysis and publication bias test were not performed [10].

## 3. Results

### 3.1. Trial Selection

**Figure 1:**
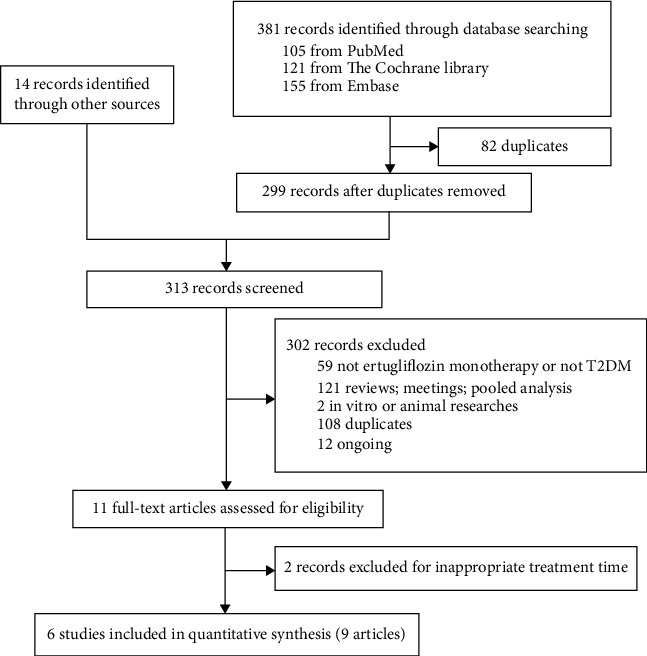
Flow chart of the literature search process.

381 articles were identified in the database retrieval. Among them, 82 articles were excluded after removing duplicates. In addition, 14 records were identified through other sources. After screening by title and abstract, 11 of the remaining 313 articles were selected for full-text assessment. Ultimately, 9 articles [6, 14–21] (6 NCT numbers) were included in the study (NCT numbers: 02630706 [14], 01999218 [15, 18], 02033889 [6, 16], 01958671 [20, 21], 02099110 [17], and 02036515 [19]). A flow chart reflecting the literature search process is shown in Figure 1.

### 3.2. Study Characteristics

This meta-analysis included six studies that were published from 2017 to 2019, enrolling 4120 participants. Three studies [17, 19–21] were conducted over 52 weeks with two 26-week periods (phase A and phase B). One study [15, 18] was conducted over 104 weeks with two 52-week periods (phase A and phase B). One study [6, 16] was conducted over 104 weeks with a 26-week period (phase A) and a 78-week period (phase B), and another study [14] was conducted over 26 weeks. Participants in one study [20, 21] were T2DM patients whose diabetes was inadequately controlled by diet and exercise. Other participants were T2DM patients whose diabetes was inadequately controlled by metformin monotherapy or combination metformin and sitagliptin. Among the six included studies, four [6, 14, 16, 19–21] compared 15 mg and 5 mg ertugliflozin monotherapy with placebo, one [15, 18] compared 15 mg and 5 mg ertugliflozin monotherapy with glimepiride, and the remaining one [17] compared 15 mg and 5 mg ertugliflozin monotherapy with sitagliptin and coadministrations. Detailed characteristics of included trials are shown in Table 1.

### 3.3. Risk of Bias Assessment

**Figure 2:**
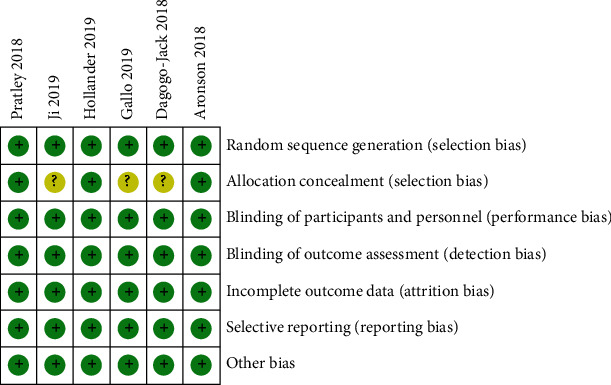
Risk of bias summary.

**Figure 3:**
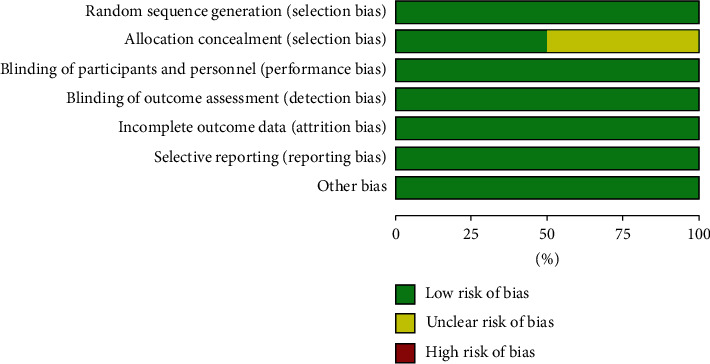
Risk of bias graph.

According to the Cochrane Risk of Bias Tool [12], three studies were assessed as low risk [15, 17, 18, 20, 21]. The randomization methods were explained in the included studies. Of these, a central electronic randomization system, an interactive automated system, and an interactive voice response system were used in the three studies, while allocation concealment of the remaining three studies was unclear. The data on ClinicalTrials.gov were also reviewed to confirm that blindness was applied during each study. The results are shown in Figures 2 and 3. Green represents a low risk of bias, yellow represents an unclear risk of bias, and red represents a high risk of bias.

### 3.4. Meta-Analysis Results

#### 3.4.1. Ertugliflozin vs. Control

**Figure 4:**
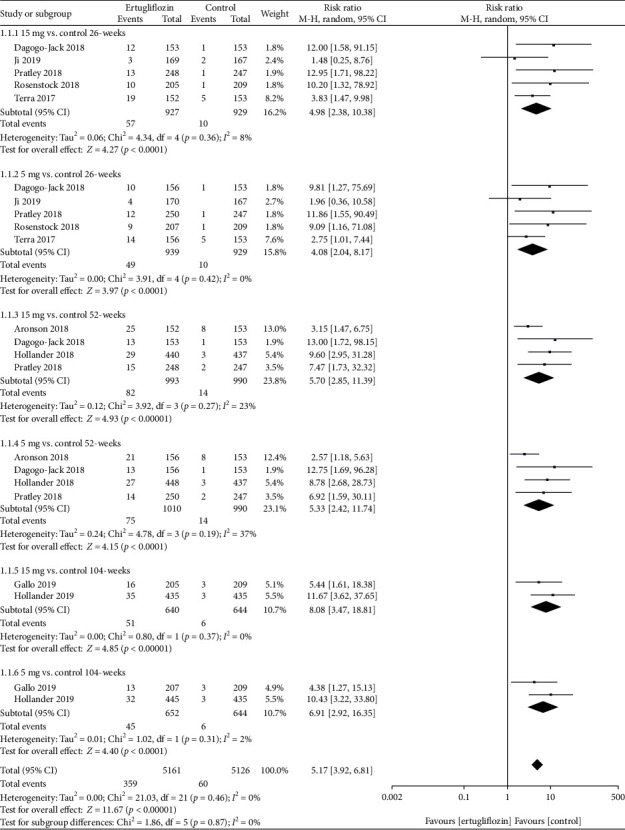
Forest plot of the risk of GMIs in the 15 mg and 5 mg dose groups compared with that in the control group at 26, 52, and 104 weeks. CI: confidence interval; M-H: Mantel-Haenszel.

*(1) Primary Outcomes*. At 26, 52, and 104 weeks, the risk of GMIs was higher in the 15 mg and 5 mg ertugliflozin groups than that in the control group (Figure 4). For GMIs, the leave-one-out sensitivity analysis showed that the results of our meta-analysis were not significantly unstable (Table 2). Considering that the control groups of Hollander et al. [18] and Pratley et al. [17] used glimepiride and sitagliptin, while other studies used placebo, we deleted both studies at 52 weeks and found that after deleting these two studies, the *p* value changed from <0.05 to >0.05 in the ertugliflozin 5 mg group.

**Figure 5:**
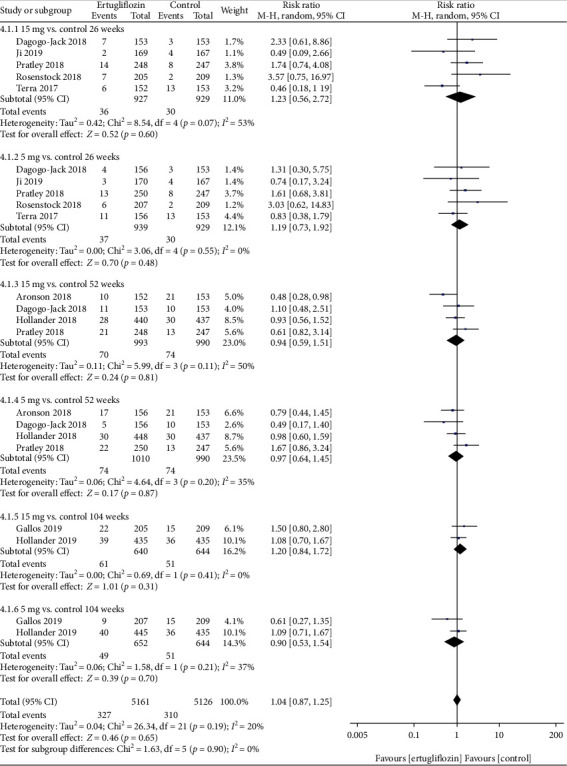
Forest plot of the risk of UTIs in the 15 mg and 5 mg dose groups compared with that in the control group at 26, 52, and 104 weeks. CI: confidence interval; M-H: Mantel-Haenszel.

No significant differences were found in the risk of UTIs at 26, 52, or 104 weeks (Figure 5). For UTIs, the leave-one-out sensitivity analysis showed that the results of our meta-analysis were not significantly unstable (Supplementary Table 1). After removing two studies that were not placebo-controlled at 52 weeks, the sensitivity analysis showed that the results of our meta-analysis were not significantly unstable.

*(2) Secondary Outcomes*. At 26 weeks, the 15 mg and 5 mg ertugliflozin groups had a higher risk of drug-related adverse events compared with the control group [(RR =1.61; 95% CI, 1.19-2.15; *p* = 0.002) and (RR =1.74; 95% CI, 1.22-2.49; *p* = 0.002), respectively] (Supplementary Figure 1). For drug-related adverse events, the leave-one-out sensitivity analysis showed that after removing Aronson et al. [20], the *p* value changed from >0.05 to <0.05 in the 15 mg ertugliflozin group at 52 weeks (Supplementary Table 2).

Compared with the control group, at 104 weeks, the risk of discontinuation related to adverse events in the 15 mg ertugliflozin group was higher (RR =1.62; 95% CI, 1.05-2.50; *p* = 0.03) (Supplementary Figure 2; sensitivity analysis was shown in Supplementary Table 3), while the risk of symptomatic hypoglycemia in the 15 mg and 5 mg ertugliflozin groups was lower [(RR =0.33; 95% CI, 0.23-0.47; *p* < 0.00001) and (RR =0.27; 95% CI, 0.11-0.66; *p* = 0.004), respectively] (Supplementary Figure 3; sensitivity analysis was shown in Supplementary Table 4). For discontinuation related to adverse events, the leave-one-out sensitivity analysis showed that after removing Gallo et al. [16] or Hollander et al. [15], the *p* value changed from <0.05 to >0.05 in the 15 mg ertugliflozin group at 104 weeks.

No significant differences were found in the risk of drug-related serious adverse events, deaths, and hypovolemia at any week (Supplementary Figures 4-6; sensitivity analysis was shown in Supplementary Tables 5 and 6).

### 3.5. GMI (Female) vs. GMI (Male)

**Figure 6:**
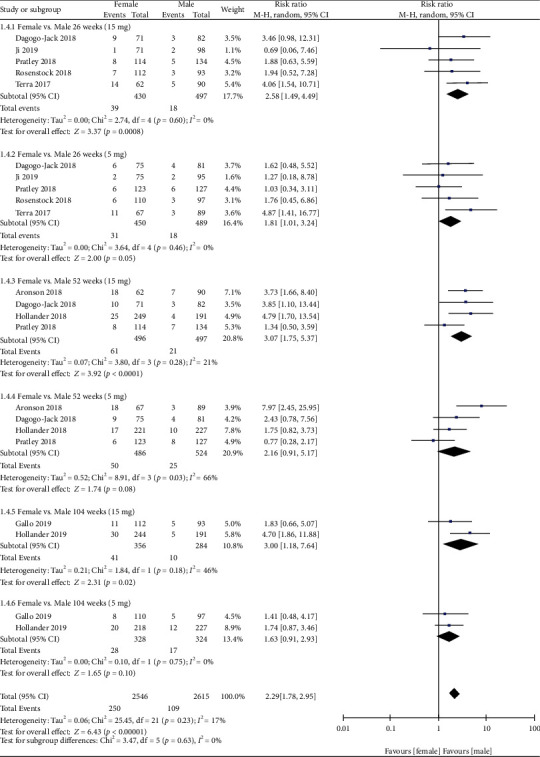
Forest plot of the risk of GMIs when comparing females with males at 26, 52, and 104 weeks. CI: confidence interval; M-H: Mantel-Haenszel.

When comparing the risk of GMIs between females and males, we found that females had a higher risk of GMIs than males in the 15 mg group at 26 weeks (RR =2.58; 95% CI, 1.49-4.49; *p* = 0.0008), 52 weeks (RR =3.07; 95% CI, 1.75-5.37; *p* < 0.0001), and 104 weeks (RR =3.00; 95% CI, 1.18-7.64; *p* = 0.02) (Figure 6). However, in the 5 mg group at 26 weeks and 52 weeks, the *p* value changed from >0.05 to <0.05 after excluding Pratley et al. [17] (sensitivity analysis was shown in Supplementary Table 7).

#### 3.5.1. Dose of 15 mg vs. That of 5 mg

**Figure 7:**
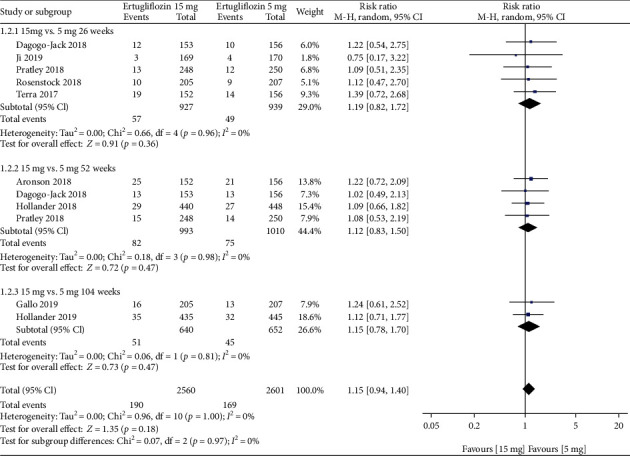
Forest plot of the risk of GMIs when comparing ertugliflozin 15 mg with 5 mg at 26, 52, and 104 weeks. CI: confidence interval; M-H: Mantel-Haenszel.

When the 15 mg group compared with the 5 mg group, no significant differences were found in the risk of GMIs (Figure 7), UTIs, drug-related adverse events, drug-related serious adverse events, discontinuation related to adverse events, deaths, symptomatic hypoglycemia, and hypovolemia in this study at either 26, 52, or 104 weeks (Supplementary Figures 7-13). We also compared the risk of GMIs between 15 mg and 5 mg groups by gender and found that there was no significant difference (Supplementary Figure 14). The leave-one-out sensitivity analysis showed that the results of our meta-analysis were not significantly unstable (sensitivity analysis was shown in Supplementary Tables 8-14).

### 3.6. Assessment of Quality of Evidence

Compared with the control group, the quality of evidence for the risk of GMIs in the 15 mg ertugliflozin group at 26, 52, and 104 weeks was all low, and the quality of evidence for the risk of GMIs in the 5 mg ertugliflozin group at 26, 52, and 104 weeks was also all low due to the small sample size, small number of included studies, and publication bias. The GRADE evidence profiles (ertugliflozin vs. control) are provided in Supplementary Tables 15 and 16.

## 4. Discussion

In this meta-analysis, we systematically reviewed current studies and found that ertugliflozin treatment had a higher risk for GMIs at every period compared with the control group. In particular, females showed a high risk for GMIs compared with males in the 15 mg ertugliflozin group. We also found that ertugliflozin decreased the risk for symptomatic hypoglycemia in a long course of treatment. There were no significant differences in the risk for UTIs, drug-related serious adverse events, deaths, or hypovolemia between the ertugliflozin group and control group, and there were also no significant differences between the two doses. In summary, a high risk for GMIs is the most prominent problem of ertugliflozin, especially among females in the high-dose group.

Diabetes patients are more susceptible to infections than nondiabetic patients. Possible causes include immune deficiency, increased adhesion of microorganisms to epithelial cells, the presence of complications, and extensive medical interventions [22]. Of the included studies, all six RCTs reported that ertugliflozin can effectively control blood glucose, reduce body weight, and improve systolic blood pressure, so the efficacy of ertugliflozin for T2DM that is otherwise inadequately controlled by conventional therapy is worthy of recognition. However, we noticed that the incidence of GMIs in the ertugliflozin group was significantly higher than that in the control group. The mechanism of SGLT2 inhibitors is considered to be the possible cause. SGLT2 inhibitors reduce blood glucose by reducing the reabsorption of glucose, so the glucose in the urine rises [23]. The increase of glucose in urine may increase the colonization rate of vaginal Candida [24] and the growth rate of urinary tract pathogens [25, 26], thereby increasing the risk of genital infections. In this study, we did not find that ertugliflozin increased the risk of UTIs compared with the control group. Among a variety of SGLT2 inhibitors, dapagliflozin produced a higher risk of UTIs than placebo and other active treatments, and it appeared to have a dose-response relationship for risk of UTIs and genital infections [27, 28]. However, patients with familial renal glucosuria rarely have UTIs [29]. It seems that urine glucose will increase the risk of GMIs, and UTIs in diabetic patients remain to be further studied [27].

A previous meta-analysis evaluated the efficacy and safety of SGLT2 inhibitors (ipragliflozin, dapagliflozin, canagliflozin, and empagliflozin), and this study showed that SGLT2 inhibitors could effectively control blood glucose, reduce body weight, and improve systolic blood pressure, but it also increased the risk of GMIs [30]. Three other meta-analyses also showed that SGLT2 inhibitors increased the risk of genital infections [27, 31, 32], and one of them showed that SGLT2 inhibitors had a net protective effect on cardiovascular outcomes and death [32]. It is widely accepted that SGLT2 inhibitors can increase the risk for GMIs, which is consistent with our findings. However, a study in China showed that empagliflozin did not increase the risk for GMIs and UTIs [33]. The safety of different SGLT2 inhibitors may be different, and the difference is worthy of further exploration. A pooled analysis from three phase III clinical trials showed that ertugliflozin had a higher rate of GMIs and drug-related adverse events, but it had no significant effect on other safety outcomes [9]. We included these studies in our analysis. Another pooled analysis of canagliflozin, dapagliflozin, and empagliflozin showed that SGLT2 inhibitors had similar relative risks in females and males, and all of them increased the risk for GMIs, but there were no sex differences [34]. This may be because there is no grouping based on the dose and follow-up time of the agent. However, a cohort study showed that female and people with prior genital infection were at higher risk of genital infections with SGLT2 inhibitor therapy [35], which is consistent with our results. Although the intensity of genital infections caused by SGLT2 inhibitors was generally mild or moderate, they tended to recur and eventually lead to treatment discontinuation and may even cause the risk of a rapid decline of renal function in some patients [26]. Therefore, genital infections should be paid attention to. In the long run, we found that ertugliflozin was not prone to cause symptomatic hypoglycemia, which may be because SGLT2 inhibitors lower blood glycemic levels independent of pancreatic *β* cell function and insulin resistance [36]. However, there were only two [15, 16] studies that reported symptomatic hypoglycemia at 104 weeks, and Gallo et al.'s [16] control group was placebo while Hollander et al.'s [15] control group was glimepiride. Glimepiride is a sulfonylurea hypoglycemic agent, and the common adverse reaction is hypoglycemia [37]. Some studies have found that the risk for hypoglycemia of glimepiride is higher than that of SGLT2 inhibitors [38]. Thus, the difference in efficacy between glimepiride and placebo may be the source of heterogeneity.

The quality of evidence in this study is from very low to low. The reason for the low level of evidence is mainly due to insufficient sample size in each period, and the interventions of the control group included in the study are not homogeneous. This is also the limitation of this research. In the future, more large-scale clinical studies need to be focused.

Based on the results of our research, in future clinical practice using ertugliflozin, we should pay special attention to high risk for GMIs, especially females on high dosages of ertugliflozin. For patients on ertugliflozin, we should advocate for early prevention of GMIs. For example, patients with a history of genital infections should closely observe the occurrence of GMIs after weighing the advantages and disadvantages, and all patients should be more vigilant with their management of genital hygiene.

## 5. Conclusion

In general, ertugliflozin is effective and tolerated for T2DM inadequately controlled by conventional therapy, but the risk for GMIs is noteworthy, especially among females in the high-dose group. However, further research is necessary to clarify the long-term safety and the potential benefits and risks of this agent.

## Figures and Tables

**Table 1:** Characteristics of studies included in the meta-analysis.

First author (year)	NCT number	Number of patients (E5/E15/C)	Baseline HbA1c	Intervention	Control	Periods
Ji (2019) [14]	NCT: 02630706	170/169/167	7.0-10.5%	1. Ertugliflozin 5 mg; 2. ertugliflozin 15 mg GRT: glimepiride	Placebo	26 weeks
Rosenstock (2018) [6], Gallo (2019) [16]	NCT: 02033889	207/205/209	7.0-10.5%	1. Ertugliflozin 5 mg; 2. ertugliflozin 15 mg GRT: glimepiride (1-26 weeks), basal insulin (27-104 weeks)	Placebo	26 weeks, 104 weeks
Hollander (2018) [18], Hollander (2019) [15]	NCT: 01999218	488/440/437	7.0-9.0%	1. Ertugliflozin 5 mg; 2. ertugliflozin 15 mg GRT: sitagliptin (1-52 weeks), not permitted (53-104 weeks)	Glimepiride up to 6 or 8 mg/d	52 weeks, 104 weeks
Pratley (2018) [17]	NCT: 02099110	250/248/247	7.5-11%	1. Ertugliflozin 5 mg; 2. ertugliflozin 15 mg GRT: glimepiride or glargine	Sitagliptin 100 mg	26 weeks, 52 weeks
Terra (2017) [21], Aronson (2018) [20]	NCT: 01958671	156/152/153	7.0-10.5%	1. Ertugliflozin 5 mg; 2. ertugliflozin 15 mg GRT: metformin (1-26 weeks), glimepiride (27-52 weeks)	Placebo (1-26 weeks), metformin (27-52 weeks)	26 weeks, 52 weeks
Dagogo-Jack (2018) [19]	NCT: 02036515	156/153/153	7.0-10.5%	1. Ertugliflozin 5 mg; 2. ertugliflozin 15 mg GRT: glimepiride or glargine	Placebo	26 weeks, 52 weeks

E5: ertugliflozin 5 mg; E15: ertugliflozin 15 mg; C: control; GRT: glycemic rescue therapy.

**Table 2:** a: Leave-one-out sensitivity analysis for GMIs (ertugliflozin vs. control). b: Sensitivity analysis by excluding two studies that were not placebo-controlled.

Study excluded	RR (95% CI)	*Z*-test *p* value	Heterogeneity (*I*^2^)
a			
15 mg vs. control 26 weeks			
Dagogo-Jack (2018) [19]	4.42 (1.96, 10.00)	*p* = 0.0004	*p* = 0.33; *I*^2^ = 12%
Ji (2019) [14]	5.98 (2.84, 12.56)	*p* < 0.00001	*p* = 0.53; *I*^2^ = 0%
Pratley (2018) [17]	4.34 (1.99, 9.47)	*p* = 0.0002	*p* = 0.36; *I*^2^ = 8%
Rosenstock (2018) [6]	4.62 (1.93, 11.03)	*p* = 0.0006	*p* = 0.29; *I*^2^ = 20%
Terra (2017) [21]	6.39 (2.12, 19.27)	*p* = 0.0010	*p* = 0.28; *I*^2^ = 21%
5 mg vs. control 26 weeks			
Dagogo-Jack (2018) [19]	3.67 (1.73, 7.77)	*p* = 0.0007	*p* = 0.38; *I*^2^ = 2%
Ji (2019) [14]	4.74 (2.21, 10.15)	*p* < 0.0001	*p* = 0.40; *I*^2^ = 0%
Pratley (2018) [17]	3.55 (1.70, 7.42)	*p* = 0.0008	*p* = 0.46; *I*^2^ = 0%
Rosenstock (2018) [6]	3.77 (1.73, 8.26)	*p* = 0.0009	*p* = 0.36; *I*^2^ = 7%
Terra (2017) [21]	5.93 (2.25, 15.58)	*p* = 0.0003	*p* = 0.46; *I*^2^ = 0%
15 mg vs. control 52 weeks			
Aronson (2018) [20]	9.32 (4.03, 21.52)	*p* < 0.00001	*p* = 0.91; *I*^2^ = 0%
Dagogo-Jack (2018) [19]	5.24 (2.44, 11.27)	*p* < 0.0001	*p* = 0.23; *I*^2^ = 33%
Hollander (2018) [18]	4.73 (2.14, 10.44)	*p* = 0.0001	*p* = 0.29; *I*^2^ = 19%
Pratley (2018) [17]	5.82 (2.27, 14.93)	*p* = 0.0002	*p* = 0.16; *I*^2^ = 45%
5 mg vs. control 52 weeks			
Aronson (2018) [20]	8.66 (3.74, 20.06)	*p* < 0.00001	*p* = 0.89; *I*^2^ = 0%
Dagogo-Jack (2018) [19]	4.73 (2.00, 11.16)	*p* = 0.0004	*p* = 0.17; *I*^2^ = 44%
Hollander (2018) [18]	4.51 (1.74, 11.70)	*p* = 0.002	*p* = 0.21; *I*^2^ = 37%
Pratley (2018) [17]	5.35 (1.85, 15.42)	*p* = 0.002	*p* = 0.11; *I*^2^ = 55%
15 mg vs. control 104 weeks			
Gallo (2019) [16]	11.67 (3.62, 37.65)	*p* < 0.0001	NA
Hollander (2019) [15]	5.44 (1.61, 18.38)	*p* = 0.006	NA
5 mg vs. control 104 weeks			
Gallo (2019) [16]	10.43 (3.22, 33.80)	*p* < 0.0001	NA
Hollander (2019) [15]	4.38 (1.27, 15.13)	*p* = 0.02	NA

b			
15 mg vs. control 52 weeks			
Hollander (2018) [18]; Pratley (2018) [17]	4.73 (1.29, 17.39)	*p* = 0.02	*p* = 0.18; *I*^2^ = 43%
5 mg vs. control 52 weeks			
Hollander (2018) [18]; Pratley (2018) [17]	4.41 (0.94, 20.64)	*p* = 0.06	*p* = 0.13; *I*^2^ = 56%

RR: risk ratio; CI: confidence interval; NA: not available.

## Data Availability

Data can be obtained from the original research articles included in this study. In addition, the data used to support the findings of this study are available from the corresponding author upon request.

## Supplementary Materials

Supplementary 1 Supplementary Figure 1: forest plot of the risk of drug-related adverse events (ertugliflozin vs. control). CI: confidence interval; M-H: Mantel-Haenszel.

Supplementary 2 Supplementary Figure 2: forest plot of the risk of discontinuation-related adverse events (ertugliflozin vs. control). CI: confidence interval; M-H: Mantel-Haenszel.

Supplementary 3 Supplementary Figure 3: Forest plot of the risk of symptomatic hypoglycemia (ertugliflozin vs. control). CI: confidence interval; M-H: Mantel-Haenszel.

Supplementary 4 Supplementary Figure 4: forest plot of the risk of drug-related serious adverse events (ertugliflozin vs. control). CI: confidence interval; M-H: Mantel-Haenszel.

Supplementary 5 Supplementary Figure 5: forest plot of the risk of deaths (ertugliflozin vs. control). CI: confidence interval; M-H: Mantel-Haenszel.

Supplementary 6 Supplementary Figure 6: forest plot of the risk of hypovolemia (ertugliflozin vs. control). CI: confidence interval; M-H: Mantel-Haenszel.

Supplementary 7 Supplementary Figure 7: forest plot the risk of UTIs (15 mg vs. 5 mg). CI: confidence interval; M-H: Mantel-Haenszel.

Supplementary 8 Supplementary Figure 8: forest plot the risk of drug-related adverse events (15 mg vs. 5 mg). CI: confidence interval; M-H: Mantel-Haenszel.

Supplementary 9 Supplementary Figure 9: forest plot the risk of drug-related serious adverse events (15 mg vs. 5 mg). CI: confidence interval; M-H: Mantel-Haenszel.

Supplementary 10 Supplementary Figure 10: forest plot the risk of discontinuation-related adverse events (15 mg vs. 5 mg). CI: confidence interval; M-H: Mantel-Haenszel.

Supplementary 11 Supplementary Figure 11: forest plot the risk of deaths (15 mg vs. 5 mg). CI: confidence interval; M-H: Mantel-Haenszel.

Supplementary 12 Supplementary Figure 12: forest plot the risk of symptomatic hypoglycemia (15 mg vs. 5 mg). CI: confidence interval; M-H: Mantel-Haenszel.

Supplementary 13 Supplementary Figure 13: forest plot the risk of hypovolemia (15 mg vs. 5 mg). CI: confidence interval; M-H: Mantel-Haenszel.

Supplementary 14 Supplementary Figure 14: forest plot of the risk of GMIs between 15 mg and 5 mg groups by gender. CI: confidence interval; M-H: Mantel-Haenszel.

Supplementary 15 Supplementary Table 1: a: leave-one-out sensitivity analysis for UTIs (ertugliflozin vs. control). b: sensitivity analysis by excluding two studies that were not placebo-controlled. RR: risk ratio; CI: confidence interval; NA: not available.

Supplementary 16 Supplementary Table 2: a: leave-one-out sensitivity analysis for drug-related adverse events (ertugliflozin vs. control). b: sensitivity analysis by excluding two studies that were not placebo-controlled. RR: risk ratio; CI: confidence interval; NA: not available.

Supplementary 17 Supplementary Table 3: a: leave-one-out sensitivity analysis for discontinuation related to adverse events (ertugliflozin vs. control). b: sensitivity analysis by excluding two studies that were not placebo-controlled. RR: risk ratio; CI: confidence interval; NA: not available.

Supplementary 18 Supplementary Table 4: a: leave-one-out sensitivity analysis for symptomatic hypoglycemia (ertugliflozin vs. control). b: sensitivity analysis by excluding two studies that were not placebo-controlled. RR: risk ratio; CI: confidence interval; NA: not available.

Supplementary 19 Supplementary Table 5: leave-one-out sensitivity analysis for deaths (ertugliflozin vs. control). RR: risk ratio; CI: confidence interval; NA: not available.

Supplementary 20 Supplementary Table 6: a: leave-one-out sensitivity analysis for hypovolemia (ertugliflozin vs. control). b: sensitivity analysis by excluding two studies that were not placebo-controlled. RR: risk ratio; CI: confidence interval; NA: not available.

Supplementary 21 Supplementary Table 7: a: leave-one-out sensitivity analysis for GMI (female vs. male). b: sensitivity analysis by excluding two studies that were not placebo-controlled. RR: risk ratio; CI: confidence interval; NA: not available.

Supplementary 22 Supplementary Table 8: leave-one-out sensitivity analysis for GMI (15 mg vs. 5 mg). RR: risk ratio; CI: confidence interval; NA: not available.

Supplementary 23 Supplementary Table 9: leave-one-out sensitivity analysis for UTI (15 mg vs. 5 mg). RR: risk ratio; CI: confidence interval; NA: not available.

Supplementary 24 Supplementary Table 10: leave-one-out sensitivity analysis for drug-related adverse events (15 mg vs. 5 mg). RR: risk ratio; CI: confidence interval; NA: not available.

Supplementary 25 Supplementary Table 11: leave-one-out sensitivity analysis for discontinuation related to adverse events (15 mg vs. 5 mg). RR: risk ratio; CI: confidence interval; NA: not available.

Supplementary 26 Supplementary Table 12: leave-one-out sensitivity analysis for deaths (15 mg vs. 5 mg). RR: risk ratio; CI: confidence interval; NA: not available.

Supplementary 27 Supplementary Table 13: leave-one-out sensitivity analysis for symptomatic hypoglycemia (15 mg vs. 5 mg). RR: risk ratio; CI: confidence interval; NA: not available.

Supplementary 28 Supplementary Table 14: leave-one-out sensitivity analysis for hypovolemia (15 mg vs. 5 mg). RR: risk ratio; CI: confidence interval; NA: not available.

Supplementary 29 Supplementary Table 15: quality of evidence for the risk of GMIs and UTIs (ertugliflozin 5 mg vs. control). High quality: we are very confident that the true effect lies close to that of the estimate of the effect. Moderate quality: we are moderately confident in the effect estimate: the true effect is likely to be close to the estimate of the effect, but there is a possibility that it is substantially different. Low quality: our confidence in the effect estimate is limited: the true effect may be substantially different from the estimate of the effect. Very low quality: we have very little confidence in the effect estimate: the true effect is likely to be substantially different from the estimate of effect. CI: confidence interval; RR: risk ratio. ^a^The sample size is small. ^b^The number of included studies is too small. ^c^All trials are funded by the pharmaceutical industry, which leads to a high risk of other biases. ^d^Point estimates vary widely from study to study. ^e^The 95% confidence interval includes no effect (i.e., confidence interval includes RR of 1.0).

Supplementary 30 Supplementary Table 16: quality of evidence for the risk of GMIs and UTIs (ertugliflozin 15 mg vs. control). High quality: we are very confident that the true effect lies close to that of the estimate of the effect. Moderate quality: we are moderately confident in the effect estimate: the true effect is likely to be close to the estimate of the effect, but there is a possibility that it is substantially different. Low quality: our confidence in the effect estimate is limited: the true effect may be substantially different from the estimate of the effect. Very low quality: we have very little confidence in the effect estimate: the true effect is likely to be substantially different from the estimate of effect. CI: confidence interval; RR: risk ratio. ^a^The sample size is small. ^b^The number of included studies is too small. ^c^All trials are funded by the pharmaceutical industry, which leads to a high risk of other biases. ^d^Point estimates vary widely from study to study. ^e^The 95% confidence interval includes no effect (i.e., confidence interval includes RR of 1.0).
