# Supplementary material for: Safety of Ertugliflozin in Patients with Type 2 Diabetes Mellitus Inadequately Controlled with Conventional Therapy at Different Periods: A Meta-Analysis of Randomized Controlled Trials
Source: J Diabetes Res. 2020 Dec 14;2020:9704659. doi: 10.1155/2020/9704659 (PMC7831274; doi:10.1155/2020/9704659)
Supplement: Supplementary 2 — Supplementary Figure 2: forest plot of the risk of discontinuation-related adverse events (ertugliflozin vs. control). CI: confidence interval; M-H: Mantel-Haenszel. [file 9704659.f2.doc]

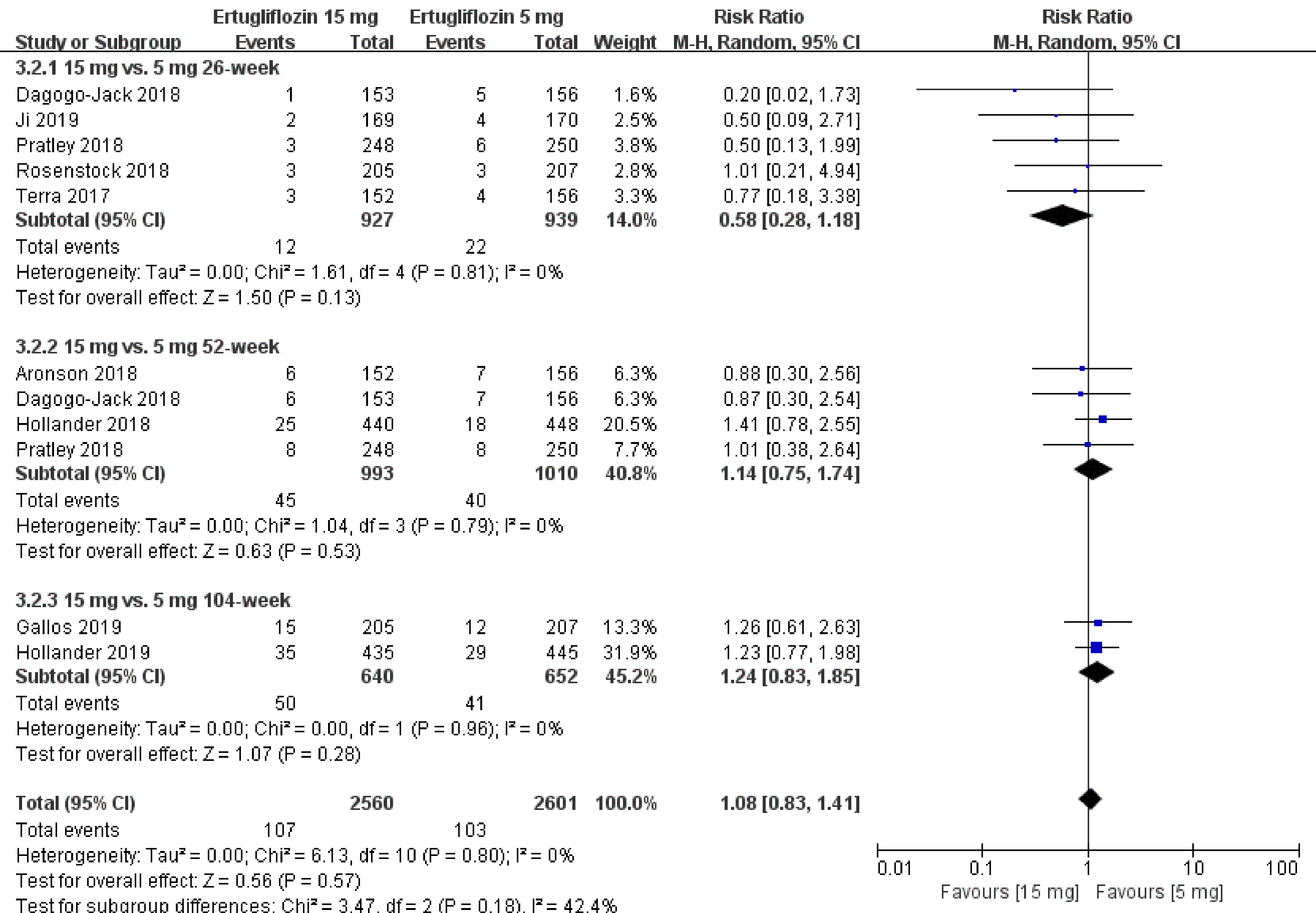


Supplementary Figure 10: Forest plot the risk of discontinuation-related adverse events (15 mg vs. 5 mg). CI: Confidence Interval; M-H: Mantel-Haensze.
