# Supplementary material for: Safety of Ertugliflozin in Patients with Type 2 Diabetes Mellitus Inadequately Controlled with Conventional Therapy at Different Periods: A Meta-Analysis of Randomized Controlled Trials
Source: J Diabetes Res. 2020 Dec 14;2020:9704659. doi: 10.1155/2020/9704659 (PMC7831274; doi:10.1155/2020/9704659)
Supplement: Supplementary 15 — Supplementary Table 1: a: leave-one-out sensitivity analysis for UTIs (ertugliflozin vs. control). b: sensitivity analysis by excluding two studies that were not placebo-controlled. RR: risk ratio; CI: confidence interval; NA: not available. [file 9704659.f15.doc]

| Study excluded | RR [95% CI] | Z-test p-value | Heterogeneity (I^2^) |
| --- | --- | --- | --- |
| a | |  |  |
| 15 mg vs. control 26-week | |  |  |
| Dagogo-Jack 2018 | 1.07 [0.42, 2.72] | p = 0.88 | p = 0.06; I² = 59% |
| Ji 2019 | 1.44 [0.60, 3.50] | p = 0.41 | p = 0.06; I² = 59% |
| Pratley 2018 | 1.11 [0.39, 3.18] | p = 0.84 | p = 0.06; I² = 59% |
| Rosenstock 2018 | 1.02 [0.44, 2.34] | p = 0.97 | p = 0.09; I² = 53% |
| Terra 2017 | 1.75 [0.93, 3.29] | p = 0.08 | p = 0.37; I² = 5% |
| 5 mg vs. control 26-week | |  |  |
| Dagogo-Jack 2018 | 1.18 [0.70, 1.96] | p = 0.54 | p = 0.39; I² = 1% |
| Ji 2019 | 1.26 [0.76, 2.09] | p = 0.38 | p = 0.46; I² = 0% |
| Pratley 2018 | 1.04 [0.58, 1.85] | p = 0.90 | p = 0.50; I² = 0% |
| Rosenstock 2018 | 1.08 [0.65, 1.79] | p = 0.76 | p = 0.66; I² = 0% |
| Terra 2017 | 1.49 [0.81, 2.75] | p = 0.20 | p = 0.64; I² = 0% |
| 15 mg vs. control 52-week | |  |  |
| Aronson 2018 | 1.12 [0.78, 1.61] | p = 0.53 | p = 0.43; I² = 0% |
| Dagogo-Jack 2018 | 0.91 [0.49, 1.68] | p = 0.75 | p = 0.05; I² = 66% |
| Hollander 2018 | 0.95 [0.46, 1.98] | p = 0.89 | p = 0.05; I² = 67% |
| Pratley 2018 | 0.80 [0.50, 1.26] | p = 0.33 | p = 0.24; I² = 30% |
| 5 mg vs. control 52-week | |  |  |
| Aronson 2018 | 1.02 [0.58, 1.80] | p = 0.93 | p = 0.14; I² = 50% |
| Dagogo-Jack 2018 | 1.06 [0.71, 1.57] | p = 0.78 | p = 0.24; I² = 29% |
| Hollander 2018 | 0.93 [0.49, 1.80] | p = 0.84 | p = 0.10; I² = 57% |
| Pratley 2018 | 0.84 [0.59, 1.20] | p = 0.33 | p = 0.50; I² = 0% |
| 15 mg vs. control 104-week | |  |  |
| Gallos 2019 | 1.08 [0.70, 1.67] | p = 0.72 | NA |
| Hollander 2019 | 1.50 [0.80, 2.80] | p = 0.21 | NA |
| 5 mg vs. control 104-week | |  |  |
| Gallos 2019 | 1.09 [0.71, 1.67] | p = 0.71 | NA |
| Hollander 2019 | 0.61 [0.27, 1.35] | p = 0.22 | NA |
| b |  |  |  |
| 15 mg vs. control 52-week | | | |
| Hollander 2018; Pratley 2018 | 0.71 [0.31, 1.59] | p = 0.40 | p = 0.14; I² = 55% |
| 5 mg vs. control 52-week | | | |
| Hollander 2018; Pratley 2018 | 0.71 [0.42, 1.19] | p = 0.19 | p = 0.43; I² = 0% |

Supplementary Table 1: a: Leave-one-out sensitivity analysis for UTIs (ertugliflozin vs. control). b: Sensitivity analysis by excluding two studies that were not placebo-controlled.

RR: Risk Ratio; CI: Confidence Interval; NA: Not Available.
