# Supplementary material for: Safety of Ertugliflozin in Patients with Type 2 Diabetes Mellitus Inadequately Controlled with Conventional Therapy at Different Periods: A Meta-Analysis of Randomized Controlled Trials
Source: J Diabetes Res. 2020 Dec 14;2020:9704659. doi: 10.1155/2020/9704659 (PMC7831274; doi:10.1155/2020/9704659)
Supplement: Supplementary 16 — Supplementary Table 2: a: leave-one-out sensitivity analysis for drug-related adverse events (ertugliflozin vs. control). b: sensitivity analysis by excluding two studies that were not placebo-controlled. RR: risk ratio; CI: confidence interval; NA: not available. [file 9704659.f16.doc]

| Study excluded | RR [95% CI] | Z-test p-value | Heterogeneity (I^2^) |
| --- | --- | --- | --- |
| 15 mg vs. 5 mg 26-week | |  |  |
| Dagogo-Jack 2018 | 0.85 [0.67, 1.08] | p = 0.19 | p = 0.74; I² = 0% |
| Ji 2019 | 0.93 [0.72, 1.18] | p = 0.54 | p = 0.41; I² = 0% |
| Pratley 2018 | 0.98 [0.76, 1.27] | p = 0.89 | p = 0.66; I² = 0% |
| Rosenstock 2018 | 0.88 [0.69, 1.12] | p = 0.29 | p = 0.45; I² = 0% |
| Terra 2017 | 0.91 [0.71, 1.17] | p = 0.46 | p = 0.39; I² = 1% |
| 15 mg vs. 5 mg 52-week | |  |  |
| Aronson 2018 | 1.15 [0.81, 1.63] | p = 0.44 | p = 0.07; I² = 62% |
| Dagogo-Jack 2018 | 0.99 [0.79, 1.25] | p = 0.96 | p = 0.25; I² = 28% |
| Hollander 2018 | 1.05 [0.70, 1.56] | p = 0.83 | p = 0.07; I² = 63% |
| Pratley 2018 | 1.18 [0.87, 1.59] | p = 0.29 | p = 0.15; I² = 48% |
| 15 mg vs. 5 mg 104-week | |  |  |
| Gallos 2019 | 1.09 [0.85, 1.38] | p = 0.51 | NA |
| Hollander 2019 | 1.31 [0.89, 1.92] | p = 0.17 | NA |

Supplementary Table 10: Leave-one-out sensitivity analysis for drug-related adverse events (15 mg vs. 5 mg).

RR: Risk Ratio; CI: Confidence Interval; NA: Not Available.
