# Supplementary material for: Safety of Ertugliflozin in Patients with Type 2 Diabetes Mellitus Inadequately Controlled with Conventional Therapy at Different Periods: A Meta-Analysis of Randomized Controlled Trials
Source: J Diabetes Res. 2020 Dec 14;2020:9704659. doi: 10.1155/2020/9704659 (PMC7831274; doi:10.1155/2020/9704659)
Supplement: Supplementary 17 — Supplementary Table 3: a: leave-one-out sensitivity analysis for discontinuation related to adverse events (ertugliflozin vs. control). b: sensitivity analysis by excluding two studies that were not placebo-controlled. RR: risk ratio; CI: confidence interval; NA: not available. [file 9704659.f17.doc]

Supplementary Table 11: Leave-one-out sensitivity analysis for discontinuation related to adverse events (15 mg vs. 5 mg).

| Study excluded | RR [95% CI] | Z-test p-value | Heterogeneity (I^2^) |
| --- | --- | --- | --- |
| 15 mg vs. 5 mg 26-week | |  |  |
| Dagogo-Jack 2018 | 0.66 [0.31, 1.41] | p = 0.28 | p = 0.90; I² = 0% |
| Ji 2019 | 0.60 [0.27, 1.32] | p = 0.20 | p = 0.66; I² = 0% |
| Pratley 2018 | 0.61 [0.26, 1.41] | p = 0.25 | p = 0.67; I² = 0% |
| Rosenstock 2018 | 0.50 [0.23, 1.12] | p = 0.09 | p = 0.80; I² = 0% |
| Terra 2017 | 0.53 [0.23, 1.20] | p = 0.13 | p = 0.70; I² = 0% |
| 15 mg vs. 5 mg 52-week | |  |  |
| Aronson 2018 | 1.20 [0.76, 1.89] | p = 0.43 | p = 0.68; I² = 0% |
| Dagogo-Jack 2018 | 1.20 [0.76, 1.90] | p = 0.43 | p = 0.69; I² = 0% |
| Hollander 2018 | 0.92 [0.51, 1.68] | p = 0.80 | p = 0.98; I² = 0% |
| Pratley 2018 | 1.18 [0.74, 1.88] | p = 0.49 | p = 0.62; I² = 0% |
| 15 mg vs. 5 mg 104-week | |  |  |
| Gallos 2019 | 1.23 [0.77, 1.98] | p = 0.38 | NA |
| Hollander 2019 | 1.26 [0.61, 2.63] | p = 0.53 | NA |

RR: Risk Ratio; CI: Confidence Interval; NA: Not Available.
