# Supplementary material for: Safety of Ertugliflozin in Patients with Type 2 Diabetes Mellitus Inadequately Controlled with Conventional Therapy at Different Periods: A Meta-Analysis of Randomized Controlled Trials
Source: J Diabetes Res. 2020 Dec 14;2020:9704659. doi: 10.1155/2020/9704659 (PMC7831274; doi:10.1155/2020/9704659)
Supplement: Supplementary 18 — Supplementary Table 4: a: leave-one-out sensitivity analysis for symptomatic hypoglycemia (ertugliflozin vs. control). b: sensitivity analysis by excluding two studies that were not placebo-controlled. RR: risk ratio; CI: confidence interval; NA: not available. [file 9704659.f18.doc]

Supplementary Table 12: Leave-one-out sensitivity analysis for deaths (15 mg vs. 5 mg).

| Study excluded | RR [95% CI] | Z-test p-value | Heterogeneity (I^2^) |
| --- | --- | --- | --- |
| 15 mg vs. 5 mg 52-week | |  |  |
| Aronson 2018 | 0.60 [0.04, 7.98] | p = 0.70 | p = 0.17; I² = 47% |
| Dagogo-Jack 2018 | - | - | - |
| Hollander 2018 | 1.02 [0.11, 9.72] | p = 0.99 | P = 0.34; I² = 0% |
| Pratley 2018 | 0.24 [0.04, 1.42] | p = 0.12 | p = 0.79; I² = 0% |
| 5 mg vs. 5 mg 104-week | |  |  |
| Gallos 2019 | 0.29 [0.06, 1.40] | p = 0.12 | NA |
| Hollander 2019 | 2.02 [0.18, 22.10] | p = 0.56 | NA |

RR: Risk Ratio; CI: Confidence Interval; NA: Not Available.
