# Supplementary material for: Safety of Ertugliflozin in Patients with Type 2 Diabetes Mellitus Inadequately Controlled with Conventional Therapy at Different Periods: A Meta-Analysis of Randomized Controlled Trials
Source: J Diabetes Res. 2020 Dec 14;2020:9704659. doi: 10.1155/2020/9704659 (PMC7831274; doi:10.1155/2020/9704659)
Supplement: Supplementary 19 — Supplementary Table 5: leave-one-out sensitivity analysis for deaths (ertugliflozin vs. control). RR: risk ratio; CI: confidence interval; NA: not available. [file 9704659.f19.doc]

| Study excluded | RR [95% CI] | Z-test p-value | Heterogeneity (I^2^) |
| --- | --- | --- | --- |
| 15 mg vs. 5 mg 26-week | |  |  |
| Dagogo-Jack 2018 | 1.32 [0.72, 2.39] | p = 0.37 | p = 0.74; I² = 0% |
| Ji 2019 | 0.93 [0.45, 1.92] | p = 0.85 | p = 0.33; I² = 13% |
| Pratley 2018 | 1.12 [0.47, 2.68] | p = 0.80 | p = 0.20; I² = 35% |
| Rosenstock 2018 | 1.12 [0.46, 2.74] | p = 0.80 | p = 0.20; I² = 35% |
| Terra 2017 | 1.01 [0.48, 2.09] | p = 0.99 | p = 0.25; I² = 27% |
| 15 mg vs. 5 mg 52-week | |  |  |
| Aronson 2018 | 1.13 [0.57, 2.26] | p = 0.72 | p = 0.20; I² = 37% |
| Dagogo-Jack 2018 | 1.54 [0.92, 2.59] | p = 0.10 | p = 0.78; I² = 0% |
| Hollander 2018 | 0.96 [0.44, 2.10] | p = 0.92 | p = 0.32; I² = 11% |
| Pratley 2018 | 1.21 [0.51, 2.87] | p = 0.66 | p = 0.18; I² = 41% |
| 15 mg vs. 5 mg 104-week | |  |  |
| Gallos 2019 | 1.68 [0.94, 3.03] | p = 0.08 | NA |
| Hollander 2019 | 1.01 [0.46, 2.20] | p = 0.98 | NA |

Supplementary Table 13: Leave-one-out sensitivity analysis for symptomatic hypoglycemia (15 mg vs. 5 mg).

RR: Risk Ratio; CI: Confidence Interval; NA: Not Available.
