# Supplementary material for: Safety of Ertugliflozin in Patients with Type 2 Diabetes Mellitus Inadequately Controlled with Conventional Therapy at Different Periods: A Meta-Analysis of Randomized Controlled Trials
Source: J Diabetes Res. 2020 Dec 14;2020:9704659. doi: 10.1155/2020/9704659 (PMC7831274; doi:10.1155/2020/9704659)
Supplement: Supplementary 20 — Supplementary Table 6: a: leave-one-out sensitivity analysis for hypovolemia (ertugliflozin vs. control). b: sensitivity analysis by excluding two studies that were not placebo-controlled. RR: risk ratio; CI: confidence interval; NA: not available. [file 9704659.f20.doc]

| Study excluded | RR [95% CI] | Z-test p-value | Heterogeneity (I^2^) |
| --- | --- | --- | --- |
| 15 mg vs. 5 mg26-week | |  |  |
| Dagogo-Jack 2018 | 1.15 [0.41, 3.21] | p = 0.80 | p = 0.66; I² = 0% |
| Ji 2019 | 0.91 [0.33, 2.56] | p = 0.86 | p = 0.66; I² = 0% |
| Pratley 2018 | 1.46 [0.44, 4.89] | p = 0.54 | p = 0.78; I² = 0% |
| Rosenstock 2018 | 0.89 [0.30, 2.61] | p = 0.83 | p = 0.63; I² = 0% |
| Terra 2017 | 0.85 [0.26, 2.77] | p = 0.79 | p = 0.62; I² = 0% |
| 15 mg vs. 5 mg 52-week | |  |  |
| Aronson 2018 | 0.53 [0.22, 1.27] | p = 0.15 | p = 0.95; I² = 0% |
| Dagogo-Jack 2018 | 0.64 [0.29, 1.41] | p = 0.27 | p = 0.79; I² = 0% |
| Hollander 2018 | 0.67 [0.27, 1.69] | p = 0.40 | p = 0.77; I² = 0% |
| Pratley 2018 | 0.64 [0.24, 1.73] | p = 0.38 | p = 0.74; I² = 0% |
| 15 mg vs. 5 mg 104-week | |  |  |
| Gallos 2019 | 1.02 [0.39, 2.70] | p = 0.96 | NA |
| Hollander 2019 | 1.01 [0.30, 3.44] | p = 0.99 | NA |

Supplementary Table 14: Leave-one-out sensitivity analysis for hypovolemia (15 mg vs. 5 mg).

RR: Risk Ratio; CI: Confidence Interval; NA: Not Available.
