# Supplementary material for: Safety of Ertugliflozin in Patients with Type 2 Diabetes Mellitus Inadequately Controlled with Conventional Therapy at Different Periods: A Meta-Analysis of Randomized Controlled Trials
Source: J Diabetes Res. 2020 Dec 14;2020:9704659. doi: 10.1155/2020/9704659 (PMC7831274; doi:10.1155/2020/9704659)
Supplement: Supplementary 21 — Supplementary Table 7: a: leave-one-out sensitivity analysis for GMI (female vs. male). b: sensitivity analysis by excluding two studies that were not placebo-controlled. RR: risk ratio; CI: confidence interval; NA: not available. [file 9704659.f21.doc]

Supplementary Table 15: Quality of evidence for the risk of GMIs and UTIs (ertugliflozin 5 mg *vs*. control).

| **Certainty assessment** | | | | | | | **№ of patients** | | **Effect** | | **Certainty** | **Importance** |
| --- | --- | --- | --- | --- | --- | --- | --- | --- | --- | --- | --- | --- |
| **№ of studies** | **Study design** | **Risk of bias** | **Inconsistency** | **Indirectness** | **Imprecision** | **Other considerations** | **Ertugliflozin 5 mg** | **control** | **Relative (95% CI)** | **Absolute (95% CI)** |
| **Genital mycotic infection (follow up: mean 26 weeks)** | | | | | | | | | | | | |
| 5 | randomised trials | not serious | not serious | not serious | serious ^a^ | publication bias strongly suspected ^b,c^ | 49/939 (5.2%) | 10/929 (1.1%) | **RR 4.08** (2.04 to 8.17) | **33 more per 1,000** (from 11 more to 77 more) | ⨁⨁◯◯ LOW | CRITICAL |
| **Genital mycotic infection (follow up: mean 52 weeks)** | | | | | | | | | | | | |
| 4 | randomised trials | not serious | not serious | not serious | serious ^a^ | publication bias strongly suspected ^b,c^ | 75/1010 (7.4%) | 14/990 (1.4%) | **RR 5.33** (2.42 to 11.74) | **61 more per 1,000** (from 20 more to 152 more) | ⨁⨁◯◯ LOW | CRITICAL |
| **Genital mycotic infection (follow up: mean 104 weeks)** | | | | | | | | | | | | |
| 2 | randomised trials | not serious | not serious | not serious | serious ^a^ | publication bias strongly suspected ^b,c^ | 45/652 (6.9%) | 6/644 (0.9%) | **RR 6.91** (2.92 to 16.35) | **55 more per 1,000** (from 18 more to 143 more) | ⨁⨁◯◯ LOW | CRITICAL |
| **Urinary tract infection (follow up: mean 26 weeks)** | | | | | | | | | | | | |
| 5 | randomised trials | not serious | serious ^d^ | not serious | serious ^a,e^ | publication bias strongly suspected ^b,c^ | 37/939 (3.9%) | 30/929 (3.2%) | **RR 1.19** (0.73 to 1.92) | **6 more per 1,000** (from 9 fewer to 30 more) | ⨁◯◯◯ VERY LOW | CRITICAL |
| **Urinary tract infection (follow up: mean 52 weeks)** | | | | | | | | | | | | |
| 4 | randomised trials | not serious | serious ^d^ | not serious | serious ^a,e^ | publication bias strongly suspected ^b,c^ | 74/1010 (7.3%) | 74/990 (7.5%) | **RR 0.97** (0.64 to 1.54) | **2 fewer per 1,000** (from 27 fewer to 40 more) | ⨁◯◯◯ VERY LOW | CRITICAL |
| **Urinary tract infection (follow up: mean 104 weeks)** | | | | | | | | | | | | |
| 2 | randomised trials | not serious | serious ^d^ | not serious | serious ^a,e^ | publication bias strongly suspected ^b,c^ | 49/652 (7.5%) | 51/644 (7.9%) | **RR 0.90** (0.53 to 1.54) | **8 fewer per 1,000** (from 37 fewer to 43 more) | ⨁◯◯◯ VERY LOW | CRITICAL |

High quality: We are very confident that the true effect lies close to that of the estimate of the effect. Moderate quality: We are moderately confident in the effect estimate: The true effect is likely to be close to the estimate of the effect, but there is a possibility that it is substantially different. Low quality: Our confidence in the effect estimate is limited: The true effect may be substantially different from the estimate of the effect. Very low quality:We have very little confidence in the effect estimate: The true effect is likely to be substantially different from the estimate of effect. CI: Confidence Interval; RR: Risk Ratio. a. The sample size is small. b. The number of included studies is too small. c. All trials are funded by the pharmaceutical industry, which leads to a high risk of other biases. d. Point estimates vary widely from study to study. e. The 95% confidence interval includes no effect (i.e. confidence interval includes RR of 1.0).
