# Supplementary material for: Safety of Ertugliflozin in Patients with Type 2 Diabetes Mellitus Inadequately Controlled with Conventional Therapy at Different Periods: A Meta-Analysis of Randomized Controlled Trials
Source: J Diabetes Res. 2020 Dec 14;2020:9704659. doi: 10.1155/2020/9704659 (PMC7831274; doi:10.1155/2020/9704659)
Supplement: Supplementary 22 — Supplementary Table 8: leave-one-out sensitivity analysis for GMI (15 mg vs. 5 mg). RR: risk ratio; CI: confidence interval; NA: not available. [file 9704659.f22.doc]

Supplementary Table 16: Quality of evidence for the risk of GMIs and UTIs (ertugliflozin 15 mg *vs*. control).

| **Certainty assessment** | | | | | | | **№ of patients** | | **Effect** | | **Certainty** | **Importance** |
| --- | --- | --- | --- | --- | --- | --- | --- | --- | --- | --- | --- | --- |
| **№ of studies** | **Study design** | **Risk of bias** | **Inconsistency** | **Indirectness** | **Imprecision** | **Other considerations** | **Ertugliflozin 15 mg** | **control** | **Relative (95% CI)** | **Absolute (95% CI)** |
| **Genital mycotic infection (follow up: mean 26 weeks)** | | | | | | | | | | | | |
| 5 | randomised trials | not serious | not serious | not serious | serious ^a^ | publication bias strongly suspected ^b,c^ | 57/927 (6.1%) | 10/929 (1.1%) | **RR 4.98** (2.38 to 10.38) | **43 more per 1,000** (from 15 more to 101 more) | ⨁⨁◯◯ LOW | CRITICAL |
| **Genital mycotic infection (follow up: mean 52 weeks)** | | | | | | | | | | | | |
| 4 | randomised trials | not serious | not serious | not serious | serious ^a^ | publication bias strongly suspected ^b,c^ | 82/993 (8.3%) | 14/990 (1.4%) | **RR 5.70** (2.85 to 11.39) | **66 more per 1,000** (from 26 more to 147 more) | ⨁⨁◯◯ LOW | CRITICAL |
| **Genital mycotic infection (follow up: mean 104 weeks)** | | | | | | | | | | | | |
| 2 | randomised trials | not serious | not serious | not serious | serious ^a^ | publication bias strongly suspected ^b,c^ | 51/640 (8.0%) | 6/644 (0.9%) | **RR 8.08** (3.47 to 18.81) | **66 more per 1,000** (from 23 more to 166 more) | ⨁⨁◯◯ LOW | CRITICAL |
| **Urinary tract infection (follow up: mean 26 weeks)** | | | | | | | | | | | | |
| 5 | randomised trials | not serious | serious ^d^ | not serious | serious ^a,e^ | publication bias strongly suspected ^b,c^ | 36/927 (3.9%) | 30/929 (3.2%) | **RR 1.23** (0.56 to 2.72) | **7 more per 1,000** (from 14 fewer to 56 more) | ⨁◯◯◯ VERY LOW | CRITICAL |
| **Urinary tract infection (follow up: mean 52 weeks)** | | | | | | | | | | | | |
| 4 | randomised trials | not serious | serious ^d^ | not serious | serious ^a,e^ | publication bias strongly suspected ^b,c^ | 70/993 (7.0%) | 74/990 (7.5%) | **RR 0.94** (0.59 to 1.51) | **4 fewer per 1,000** (from 31 fewer to 38 more) | ⨁◯◯◯ VERY LOW | CRITICAL |
| **Urinary tract infection (follow up: mean 104 weeks)** | | | | | | | | | | | | |
| 2 | randomised trials | not serious | not serious | not serious | serious ^a,e^ | publication bias strongly suspected ^b,c^ | 61/640 (9.5%) | 51/644 (7.9%) | **RR 1.20** (0.84 to 1.72) | **16 more per 1,000** (from 13 fewer to 57 more) | ⨁⨁◯◯ LOW | CRITICAL |

High quality: We are very confident that the true effect lies close to that of the estimate of the effect. Moderate quality: We are moderately confident in the effect estimate: The true effect is likely to be close to the estimate of the effect, but there is a possibility that it is substantially different. Low quality: Our confidence in the effect estimate is limited: The true effect may be substantially different from the estimate of the effect. Very low quality:We have very little confidence in the effect estimate: The true effect is likely to be substantially different from the estimate of effect. CI: Confidence Interval; RR: Risk Ratio. a. The sample size is small. b. The number of included studies is too small. c. All trials are funded by the pharmaceutical industry, which leads to a high risk of other biases. d. Point estimates vary widely from study to study. e. The 95% confidence interval includes no effect (i.e. confidence interva includes RR of 1.0).
