# Supplementary material for: Safety of Ertugliflozin in Patients with Type 2 Diabetes Mellitus Inadequately Controlled with Conventional Therapy at Different Periods: A Meta-Analysis of Randomized Controlled Trials
Source: J Diabetes Res. 2020 Dec 14;2020:9704659. doi: 10.1155/2020/9704659 (PMC7831274; doi:10.1155/2020/9704659)
Supplement: Supplementary 23 — Supplementary Table 9: leave-one-out sensitivity analysis for UTI (15 mg vs. 5 mg). RR: risk ratio; CI: confidence interval; NA: not available. [file 9704659.f23.doc]

| Study excluded | RR [95% CI] | Z-test p-value | Heterogeneity (I^2^) |
| --- | --- | --- | --- |
| a | |  |  |
| 15 mg vs. control 26-week | |  |  |
| Dagogo-Jack 2018 | 1.60 [1.10, 2.32] | p = 0.01 | p = 0.18; I² = 39% |
| Ji 2019 | 1.83 [1.35, 2.48] | p = 0.0001 | p = 0.67; I² = 0% |
| Pratley 2018 | 1.45 [1.08, 1.93] | p = 0.01 | p = 0.45; I² = 0% |
| Rosenstock 2018 | 1.54 [1.08, 2.20] | p = 0.02 | p = 0.22; I² = 32% |
| Terra 2017 | 1.66 [1.12, 2.45] | p = 0.01 | p = 0.18; I² = 38% |
| 5 mg vs. control 26-week | |  |  |
| Dagogo-Jack 2018 | 1.86 [1.22, 2.83] | p = 0.004 | p = 0.09; I² = 55% |
| Ji 2019 | 1.93 [1.29, 2.89] | p = 0.001 | p = 0.16; I² = 42% |
| Pratley 2018 | 1.47 [1.11, 1.96] | p = 0.008 | p = 0.73; I² = 0% |
| Rosenstock 2018 | 1.72 [1.10, 2.70] | p = 0.02 | p = 0.06; I² = 59% |
| Terra 2017 | 1.78 [1.10, 2.86] | p = 0.02 | p = 0.06; I² = 59% |
| 15 mg vs. control 52-week | |  |  |
| Aronson 2018 | 1.49 [1.09, 2.04] | p = 0.01 | p = 0.19; I² = 40% |
| Dagogo-Jack 2018 | 1.20 [0.81, 1.80] | p = 0.36 | p = 0.03; I² = 72% |
| Hollander 2018 | 1.38 [0.77, 2.44] | p = 0.28 | p = 0.01; I² = 78% |
| Pratley 2018 | 1.17 [0.81, 1.69] | p = 0.40 | p = 0.06; I² = 65% |
| 5 mg vs. control 52-week | |  |  |
| Aronson 2018 | 1.34 [0.79, 2.27] | p = 0.28 | p = 0.01; I² = 77% |
| Dagogo-Jack 2018 | 1.25 [0.78, 2.02] | p = 0.36 | p = 0.006; I² = 81% |
| Hollander 2018 | 1.29 [0.71, 2.36] | p = 0.40 | p = 0.008; I² = 79% |
| Pratley 2018 | 0.99 [0.80, 1.22] | p = 0.91 | p = 0.87; I² = 0% |
| 15 mg vs. control 104-week | |  |  |
| Gallos 2019 | 1.13 [0.88, 1.45] | p = 0.33 | NA |
| Hollander 2019 | 1.00 [0.70, 1.41] | p = 0.99 | NA |
| 5 mg vs. control 104-week | |  |  |
| Gallos 2019 | 1.04 [0.81, 1.34] | p = 0.75 | NA |
| Hollander 2019 | 0.76 [0.52, 1.12] | p = 0.16 | NA |
| b |  |  |  |
| 15 mg vs. control 52-week | | | |
| Hollander 2018; Pratley 2018 | 1.18 [0.56, 2.51] | p = 0.66 | p = 0.02; I² = 81% |
| 5 mg vs. control 52-week | | | |
| Hollander 2018; Pratley 2018 | 0.94 [0.70, 1.28] | p = 0.72 | p = 0.73; I² = 0% |

Supplementary Table 2: a: Leave-one-out sensitivity analysis for drug-related adverse events (ertugliflozin vs. control). b: Sensitivity analysis by excluding two studies that were not placebo-controlled.

RR: Risk Ratio; CI: Confidence Interval; NA: Not Available.
