# Supplementary material for: Safety of Ertugliflozin in Patients with Type 2 Diabetes Mellitus Inadequately Controlled with Conventional Therapy at Different Periods: A Meta-Analysis of Randomized Controlled Trials
Source: J Diabetes Res. 2020 Dec 14;2020:9704659. doi: 10.1155/2020/9704659 (PMC7831274; doi:10.1155/2020/9704659)
Supplement: Supplementary 24 — Supplementary Table 10: leave-one-out sensitivity analysis for drug-related adverse events (15 mg vs. 5 mg). RR: risk ratio; CI: confidence interval; NA: not available. [file 9704659.f24.doc]

| Study excluded | RR [95% CI] | Z-test p-value | Heterogeneity (I^2^) |
| --- | --- | --- | --- |
| a | |  |  |
| 15 mg vs. control 26-week | |  |  |
| Dagogo-Jack 2018 | 0.82 [0.36, 1.89] | p = 0.65 | p = 0.60; I² = 0% |
| Ji 2019 | 0.98 [0.40, 2.41] | p = 0.96 | p = 0.71; I² = 0% |
| Pratley 2018 | 0.70 [0.30, 1.64] | p = 0.41 | p = 0.92; I² = 0% |
| Rosenstock 2018 | 0.78 [0.31, 1.97] | p = 0.61 | p = 0.61; I² = 0% |
| Terra 2017 | 0.98 [0.37, 2.56] | p = 0.96 | p = 0.66; I² = 0% |
| 5 mg vs. control 26-week | |  |  |
| Dagogo-Jack 2018 | 1.15 [0.54, 2.46] | p = 0.71 | p = 0.42; I² = 0% |
| Ji 2019 | 1.69 [0.62, 4.62] | p = 0.31 | p = 0.25; I² = 28% |
| Pratley 2018 | 1.12 [0.53, 2.39] | p = 0.77 | p = 0.53; I² = 0% |
| Rosenstock 2018 | 1.63 [0.61, 4.34] | p = 0.33 | p = 0.23; I² = 30% |
| Terra 2017 | 1.78 [0.71, 4.48] | p = 0.22 | p = 0.33; I² = 12% |
| 15 mg vs. control 52-week | |  |  |
| Aronson 2018 | 1.29 [0.81, 2.06] | p = 0.28 | p = 0.81; I² = 0% |
| Dagogo-Jack 2018 | 1.13 [0.69, 1.85] | p = 0.64 | p = 0.33; I² = 11% |
| Hollander 2018 | 0.87 [0.48, 1.58] | p = 0.65 | p = 0.65; I² = 0% |
| Pratley 2018 | 1.09 [0.65, 1.83] | p = 0.74 | p = 0.32; I² = 13% |
| 5 mg vs. control 52-week | |  |  |
| Aronson 2018 | 1.08 [0.66, 1.75] | p = 0.76 | p = 0.98; I² = 0% |
| Dagogo-Jack 2018 | 0.95 [0.59, 1.52] | p = 0.83 | p = 0.73; I² = 0% |
| Hollander 2018 | 0.94 [0.53, 1.67] | p = 0.83 | p = 0.71; I² = 0% |
| Pratley 2018 | 0.95 [0.59, 1.53] | p = 0.83 | p = 0.73; I² = 0% |
| 15 mg vs. control 104-week | |  |  |
| Gallos 2019 | 1.59 [0.95, 2.67] | p = 0.08 | NA |
| Hollander 2019 | 1.70 [0.76, 3.80] | p = 0.20 | NA |
| 5 mg vs. control 104-week | |  |  |
| Gallos 2019 | 1.29 [0.75, 2.21] | p = 0.36 | NA |
| Hollander 2019 | 1.35 [0.58, 3.13] | p = 0.49 | NA |
| b |  |  |  |
| 15 mg vs. control 52-week | | | |
| Hollander 2018; Pratley 2018 | 0.75 [0.36, 1.58] | p = 0.45 | p = 0.51; I² = 0% |
| 5 mg vs. control 52-week | | | |
| Hollander 2018; Pratley 2018 | 0.86 [0.42, 1.74] | p = 0.67 | p = 0.48; I² = 0% |

Supplementary Table 3: a: Leave-one-out sensitivity analysis for discontinuation related to adverse events (ertugliflozin vs. control). b: Sensitivity analysis by excluding two studies that were not placebo-controlled.

RR: Risk Ratio; CI: Confidence Interval; NA: Not Available.
