# Supplementary material for: Safety of Ertugliflozin in Patients with Type 2 Diabetes Mellitus Inadequately Controlled with Conventional Therapy at Different Periods: A Meta-Analysis of Randomized Controlled Trials
Source: J Diabetes Res. 2020 Dec 14;2020:9704659. doi: 10.1155/2020/9704659 (PMC7831274; doi:10.1155/2020/9704659)
Supplement: Supplementary 25 — Supplementary Table 11: leave-one-out sensitivity analysis for discontinuation related to adverse events (15 mg vs. 5 mg). RR: risk ratio; CI: confidence interval; NA: not available. [file 9704659.f25.doc]

Supplementary Table 4: a: Leave-one-out sensitivity analysis for symptomatic hypoglycemia (ertugliflozin vs. control). b: Sensitivity analysis by excluding two studies that were not placebo-controlled.

| Study excluded | RR [95% CI] | Z-test p-value | Heterogeneity (I^2^) |
| --- | --- | --- | --- |
| a | |  |  |
| 15 mg vs. control 26-week | |  |  |
| Dagogo-Jack 2018 | 1.74 [0.85, 3.56] | p = 0.13 | p = 0.37; I² = 5% |
| Ji 2019 | 1.18 [0.59, 2.38] | p = 0.64 | p = 0.41; I² = 0% |
| Pratley 2018 | 1.72 [0.56, 5.32] | p = 0.35 | p = 0.16; I² = 42% |
| Rosenstock 2018 | 1.39 [0.43, 4.46] | p = 0.58 | p = 0.13; I² = 47% |
| Terra 2017 | 1.38 [0.48, 3.96] | p = 0.55 | p = 0.13; I² = 47% |
| 5 mg vs. control 26-week | |  |  |
| Dagogo-Jack 2018 | 1.40 [0.69, 2.87] | p = 0.35 | p= 0.68; I² = 0% |
| Ji 2019 | 1.30 [0.68, 2.48] | p = 0.43 | p = 0.90; I² = 0% |
| Pratley 2018 | 1.67 [0.79, 3.51] | p = 0.18 | p = 0.82; I² = 0% |
| Rosenstock 2018 | 1.31 [0.64, 2.70] | p = 0.46 | p = 0.72; I² = 0% |
| Terra 2017 | 1.48 [0.77, 2.85] | p = 0.24 | p = 0.71; I² = 0% |
| 15 mg vs. control 52-week | |  |  |
| Aronson 2018 | 0.50 [0.19, 1.30] | p = 0.16 | p = 0.03; I² = 71% |
| Dagogo-Jack 2018 | 0.52 [0.20, 1.33] | p = 0.17 | p = 0.03; I² = 72% |
| Hollander 2018 | 0.76 [0.39, 1.48] | p = 0.42 | p = 0.55; I² = 0% |
| Pratley 2018 | 0.31 [0.21, 0.46] | p < 0.00001 | p = 0.40; I² = 0% |
| 5 mg vs. control 52-week | |  |  |
| Aronson 2018 | 0.54 [0.13, 2.24] | p = 0.39 | p = 0.0003; I² = 88% |
| Dagogo-Jack 2018 | 0.34 [0.10, 1.18] | p = 0.09 | p = 0.010; I² = 78% |
| Hollander 2018 | 0.81 [0.39, 1.69] | p = 0.58 | p = 0.31; I² = 15% |
| Pratley 2018 | 0.36 [0.10, 1.35] | p = 0.13 | p = 0.006; I² = 80% |
| 15 mg vs. control 104-week | |  |  |
| Gallos 2019 | 0.29 [0.20, 0.43] | p < 0.00001 | NA |
| Hollander 2019 | 0.44 [0.23, 0.84] | p = 0.01 | NA |
| 5 mg vs. control 104-week | |  |  |
| Gallos 2019 | 0.17 [0.11, 0.28] | p < 0.00001 | NA |
| Hollander 2019 | 0.43 [0.23, 0.83] | p = 0.01 | NA |
| b |  |  |  |
| 15 mg vs. control 52-week | | | |
| Hollander 2018; Pratley 2018 | 0.54 [0.22, 1.34] | p = 0.18 | p = 0.88; I² = 0% |
| 5 mg vs. control 52-week | | | |
| Hollander 2018; Pratley 2018 | 0.64 [0.16, 2.50] | p = 0.52 | p = 0.14; I² = 54% |

RR: Risk Ratio; CI: Confidence Interval; NA: Not Available.
