# Supplementary material for: Safety of Ertugliflozin in Patients with Type 2 Diabetes Mellitus Inadequately Controlled with Conventional Therapy at Different Periods: A Meta-Analysis of Randomized Controlled Trials
Source: J Diabetes Res. 2020 Dec 14;2020:9704659. doi: 10.1155/2020/9704659 (PMC7831274; doi:10.1155/2020/9704659)
Supplement: Supplementary 26 — Supplementary Table 12: leave-one-out sensitivity analysis for deaths (15 mg vs. 5 mg). RR: risk ratio; CI: confidence interval; NA: not available. [file 9704659.f26.doc]

| Study excluded | RR [95% CI] | Z-test p-value | Heterogeneity (I^2^) |
| --- | --- | --- | --- |
| 15 mg vs. control 52-week | |  |  |
| Aronson 2018 | - | - | - |
| Dagogo-Jack 2018 | - | - | - |
| Hollander 2018 | 2.99 [0.12, 72.99] | p = 0.50 | NA |
| Pratley 2018 | 2.98 [0.12, 72.94] | p = 0.50 | NA |
| 5 mg vs. control 52-week | |  |  |
| Aronson 2018 | 10.73 [0.60, 193.48] | p = 0.11 | NA |
| Dagogo-Jack 2018 | - | - | - |
| Hollander 2018 | 2.94 [0.12, 71.68] | p = 0.51 | NA |
| Pratley 2018 | - | - | - |
| 15 mg vs. control 104-week | |  |  |
| Gallos 2019 | 2.00 [0.18, 21.98] | p = 0.57 | NA |
| Hollander 2019 | 0.68 [0.11, 4.03] | p = 0.67 | NA |
| 5 mg vs. control 104-week | |  |  |
| Gallos 2019 | 6.84 [0.85, 55.38] | p = 0.07 | NA |
| Hollander 2019 | 0.34 [0.04, 3.21] | p = 0.34 | NA |

Supplementary Table 5: Leave-one-out sensitivity analysis for deaths (ertugliflozin vs. control).

RR: Risk Ratio; CI: Confidence Interval; NA: Not Available.
