# Supplementary material for: Safety of Ertugliflozin in Patients with Type 2 Diabetes Mellitus Inadequately Controlled with Conventional Therapy at Different Periods: A Meta-Analysis of Randomized Controlled Trials
Source: J Diabetes Res. 2020 Dec 14;2020:9704659. doi: 10.1155/2020/9704659 (PMC7831274; doi:10.1155/2020/9704659)
Supplement: Supplementary 27 — Supplementary Table 13: leave-one-out sensitivity analysis for symptomatic hypoglycemia (15 mg vs. 5 mg). RR: risk ratio; CI: confidence interval; NA: not available. [file 9704659.f27.doc]

Supplementary Table 6: a: Leave-one-out sensitivity analysis for hypovolemia (ertugliflozin vs. control). b: Sensitivity analysis by excluding two studies that were not placebo-controlled.

| Study excluded | RR [95% CI] | Z-test p-value | Heterogeneity (I^2^) |
| --- | --- | --- | --- |
| a | |  |  |
| 15 mg vs. control 26-week | |  |  |
| Dagogo-Jack 2018 | 0.93 [0.33, 2.59] | p = 0.89 | p = 0.50; I² = 0% |
| Ji 2019 | 0.83 [0.29, 2.35] | p = 0.72 | p = 0.43; I² = 0% |
| Pratley 2018 | 0.69 [0.24, 1.93] | p = 0.48 | p = 0.74; I² = 0% |
| Rosenstock 2018 | 0.71 [0.24, 2.06] | p = 0.53 | p = 0.55; I² = 0% |
| Terra 2017 | 1.45 [0.36, 5.85] | p = 0.60 | p = 0.66; I² = 0% |
| 5 mg vs. control 26-week | |  |  |
| Dagogo-Jack 2018 | 0.79 [0.18, 3.49] | p = 0.76 | p = 0.23; I² = 30% |
| Ji 2019 | 0.93 [0.23, 3.71] | p = 0.92 | p = 0.26; I² = 26% |
| Pratley 2018 | 0.48 [0.15, 1.51] | p = 0.21 | p = 0.85; I² = 0% |
| Rosenstock 2018 | 0.79 [0.18, 3.46] | p = 0.75 | p = 0.23; I² = 30% |
| Terra 2017 | 1.36 [0.32, 5.77] | p = 0.68 | p = 0.46; I² = 0% |
| 15 mg vs. control 52-week | |  |  |
| Aronson 2018 | 1.15 [0.29, 4.62] | p = 0.85 | p = 0.28; I² = 22% |
| Dagogo-Jack 2018 | 0.94 [0.29, 3.01] | p = 0.92 | p = 0.23; I² = 33% |
| Hollander 2018 | 0.74 [0.15, 3.66] | p = 0.71 | p = 0.16; I² = 45% |
| Pratley 2018 | 0.54 [0.21, 1.43] | p = 0.22 | p = 0.58; I² = 0% |
| 5 mg vs. control 52-week | |  |  |
| Aronson 2018 | 2.03 [0.58, 7.20] | p = 0.27 | p = 0.26; I² = 27% |
| Dagogo-Jack 2018 | 1.54 [0.34, 6.98] | p = 0.58 | p = 0.06; I² = 64% |
| Hollander 2018 | 1.05 [0.17, 6.38] | p = 0.96 | p = 0.07; I² = 63% |
| Pratley 2018 | 0.80 [0.28, 2.31] | p = 0.69 | p = 0.26; I² = 25% |
| 15 mg vs. control 104-week | |  |  |
| Gallos 2019 | 2.67 [0.71, 9.98] | p = 0.15 | NA |
| Hollander 2019 | 1.70 [0.41, 7.02] | p = 0.46 | NA |
| 5 mg vs. control 104-week | |  |  |
| Gallos 2019 | 2.61 [0.70, 9.76] | p = 0.15 | NA |
| Hollander 2019 | 1.68 [0.41, 6.95] | p = 0.47 | NA |
| a |  |  |  |
| 15 mg vs. control 52-week | | | |
| Hollander 2018; Pratley 2018 | 0.38 [0.11, 1.29] | p = 0.12 | p = 0.65; I² = 0% |
| 5 mg vs. control 52-week | | | |
| Hollander 2018; Pratley 2018 | 0.44 [0.14, 1.40] | p = 0.16 | p = 0.91; I² = 0% |

RR: Risk Ratio; CI: Confidence Interval; NA: Not Available.
