# Supplementary material for: Safety of Ertugliflozin in Patients with Type 2 Diabetes Mellitus Inadequately Controlled with Conventional Therapy at Different Periods: A Meta-Analysis of Randomized Controlled Trials
Source: J Diabetes Res. 2020 Dec 14;2020:9704659. doi: 10.1155/2020/9704659 (PMC7831274; doi:10.1155/2020/9704659)
Supplement: Supplementary 28 — Supplementary Table 14: leave-one-out sensitivity analysis for hypovolemia (15 mg vs. 5 mg). RR: risk ratio; CI: confidence interval; NA: not available. [file 9704659.f28.doc]

| Study excluded | RR [95% CI] | Z-test p-value | Heterogeneity (I^2^) |
| --- | --- | --- | --- |
| a | |  |  |
| Female vs. Male 26-week (15 mg) | |  |  |
| Dagogo-Jack 2018 | 2.41 [1.31, 4.46] | p = 0.005 | p = 0.48; I² = 0% |
| Ji 2019 | 2.79 [1.58, 4.92] | p = 0.0004 | p = 0.69; I² = 0% |
| Pratley 2018 | 2.89 [1.52, 5.48] | p = 0.001 | p = 0.51; I² = 0% |
| Rosenstock 2018 | 2.75 [1.50, 5.04] | p = 0.001 | p = 0.47; I² = 0% |
| Terra 2017 | 2.08 [1.06, 4.07] | p = 0.03 | p = 0.68; I² = 0% |
| Female vs. Male 26-week (5 mg) | |  |  |
| Dagogo-Jack 2018 | 1.88 [0.90, 3.92] | p = 0.09 | p = 0.31; I² = 17% |
| Ji 2019 | 1.89 [0.98, 3.66] | p = 0.06 | p = 0.32; I² = 14% |
| Pratley 2018 | 2.25 [1.13, 4.47] | p = 0.02 | p = 0.52; I² = 0% |
| Rosenstock 2018 | 1.83 [0.89, 3.76] | p = 0.10 | p = 0.30; I² = 18% |
| Terra 2017 |  |  |  |
| Female vs. Male 52-week (15 mg) | |  |  |
| Aronson 2018 | 2.81 [1.23, 6.43] | p = 0.01 | p = 0.17; I² = 43% |
| Dagogo-Jack 2018 | 2.91 [1.40, 6.06] | p = 0.004 | p = 0.16; I² = 45% |
| Hollander 2018 | 2.67 [1.35, 5.29] | p = 0.005 | p = 0.24; I² = 30% |
| Pratley 2018 | 4.05 [2.29, 7.15] | p < 0.00001 | p = 0.93; I² = 0% |
| Female vs. Male 52-week (5 mg) | |  |  |
| Aronson 2018 | 1.50 [0.82, 2.74] | p = 0.19 | p = 0.30; I² = 18% |
| Dagogo-Jack 2018 | 2.12 [0.65, 6.96] | p = 0.21 | p = 0.01; I² = 77% |
| Hollander 2018 | 2.41 [0.63, 9.21] | p = 0.20 | p = 0.01; I² = 77% |
| Pratley 2018 | 2.99 [1.23, 7.27] | p = 0.02 | p = 0.10; I² = 57% |
| Female vs. Male 104-week (15 mg) | |  |  |
| Gallos 2019 | 4.70 [1.86, 11.88] | p = 0.001 | NA |
| Hollander 2019 | 1.83 [0.66, 5.07] | p = 0.25 | NA |
| Female vs. Male 104-week (5 mg) | |  |  |
| Gallos 2019 | 1.74 [0.87, 3.46] | p = 0.12 | NA |
| Hollander 2019 | 1.41 [0.48, 4.17] | p = 0.53 | NA |
| b |  |  |  |
| Female vs. Male 52-week (15 mg) | | | |
| Hollander 2018; Pratley 2018 | 3.77 [1.91, 7.44] | p = 0.0001 | p = 0.97; I² = 0% |
| Female vs. Male 52-week (5 mg) | | | |
| Hollander 2018; Pratley 2018 | 4.35 [1.35, 14.08] | p = 0.01 | p = 0.15; I² = 51% |

Supplementary Table 7: a: Leave-one-out sensitivity analysis for GMI (female vs. male). b: Sensitivity analysis by excluding two studies that were not placebo-controlled.

RR: Risk Ratio; CI: Confidence Interval; NA: Not Available.
