# Supplementary material for: Safety of Ertugliflozin in Patients with Type 2 Diabetes Mellitus Inadequately Controlled with Conventional Therapy at Different Periods: A Meta-Analysis of Randomized Controlled Trials
Source: J Diabetes Res. 2020 Dec 14;2020:9704659. doi: 10.1155/2020/9704659 (PMC7831274; doi:10.1155/2020/9704659)
Supplement: Supplementary 29 — Supplementary Table 15: quality of evidence for the risk of GMIs and UTIs (ertugliflozin 5 mg vs. control). High quality: we are very confident that the true effect lies close to that of the estimate of the effect. Moderate quality: we are moderately confident in the effect estimate: the true effect is likely to be close to the estimate of the effect, but there is a possibility that it is substantially different. Low quality: our confidence in the effect estimate is limited: the true effect may be substantially different from the estimate of the effect. Very low quality: we have very little confidence in the effect estimate: the true effect is likely to be substantially different from the estimate of effect. CI: confidence interval; RR: risk ratio. aThe sample size is small. bThe number of included studies is too small. cAll trials are funded by the pharmaceutical industry, which leads to a high risk of other biases. dPoint estimates vary widely from study to study. eThe 95% confidence interval includes no effect (i.e., confidence interval includes RR of 1.0). [file 9704659.f29.doc]

Supplementary Table 8: Leave-one-out sensitivity analysis for GMI (15 mg vs. 5 mg).

| Study excluded | RR [95% CI] | Z-test p-value | Heterogeneity (I^2^) |
| --- | --- | --- | --- |
| 15 mg vs. 5mg 26-week | |  |  |
| Dagogo-Jack 2018 | 1.18 [0.78, 1.78] | p = 0.44 | p = 0.88; I² = 0% |
| Ji 2019 | 1.22 [0.84, 1.79] | p = 0.30 | p = 0.96; I² = 0% |
| Pratley 2018 | 1.22 [0.80, 1.86] | p = 0.36 | p = 0.90; I² = 0% |
| Rosenstock 2018 | 1.20 [0.80, 1.80] | p = 0.38 | p = 0.89; I² = 0% |
| Terra 2017 | 1.10 [0.70, 1.72] | p= 0.67 | p = 0.96; I² = 0% |
| 15 mg vs. 5 mg 52-week | |  |  |
| Aronson 2018 | 1.07 [0.75, 1.54] | p = 0.70 | p= 0.99; I² = 0% |
| Dagogo-Jack 2018 | 1.14 [0.82, 1.58] | p = 0.44 | p= 0.95; I² = 0% |
| Hollander 2018 | 1.13 [0.78, 1.63] | p = 0.52 | p = 0.92; I² = 0% |
| Pratley 2018 | 1.12 [0.81, 1.56] | p = 0.48 | p = 0.92; I² = 0% |
| 15 mg vs. 5 mg 104-week | |  |  |
| Gallos 2019 | 1.12 [0.71, 1.77] | p = 0.63 | NA |
| Hollander 2019 | 1.24 [0.61, 2.52] | p = 0.55 | NA |

RR: Risk Ratio; CI: Confidence Interval; NA: Not Available.
