# Supplementary material for: Safety of Ertugliflozin in Patients with Type 2 Diabetes Mellitus Inadequately Controlled with Conventional Therapy at Different Periods: A Meta-Analysis of Randomized Controlled Trials
Source: J Diabetes Res. 2020 Dec 14;2020:9704659. doi: 10.1155/2020/9704659 (PMC7831274; doi:10.1155/2020/9704659)
Supplement: Supplementary 30 — Supplementary Table 16: quality of evidence for the risk of GMIs and UTIs (ertugliflozin 15 mg vs. control). High quality: we are very confident that the true effect lies close to that of the estimate of the effect. Moderate quality: we are moderately confident in the effect estimate: the true effect is likely to be close to the estimate of the effect, but there is a possibility that it is substantially different. Low quality: our confidence in the effect estimate is limited: the true effect may be substantially different from the estimate of the effect. Very low quality: we have very little confidence in the effect estimate: the true effect is likely to be substantially different from the estimate of effect. CI: confidence interval; RR: risk ratio. aThe sample size is small. bThe number of included studies is too small. cAll trials are funded by the pharmaceutical industry, which leads to a high risk of other biases. dPoint estimates vary widely from study to study. eThe 95% confidence interval includes no effect (i.e., confidence interval includes RR of 1.0). [file 9704659.f30.doc]

Supplementary Table 9: Leave-one-out sensitivity analysis for UTI (15 mg vs. 5 mg).

| Study excluded | RR [95% CI] | Z-test p-value | Heterogeneity (I^2^) |
| --- | --- | --- | --- |
| 15 mg vs. 5 mg 26-week | |  |  |
| Dagogo-Jack 2018 | 0.90 [0.55, 1.47] | p = 0.66 | p = 0.68; I² = 0% |
| Ji 2019 | 1.02 [0.63, 1.63] | p = 0.95 | p = 0.50; I² = 0% |
| Pratley 2018 | 0.93 [0.52, 1.67] | p = 0.81 | p = 0.48; I² = 0% |
| Rosenstock 2018 | 0.95 [0.57, 1.58] | p = 0.85 | p = 0.48; I² = 0% |
| Terra 2017 | 1.16 [0.69, 1.95] | p = 0.57 | p = 0.83; I² = 0% |
| 15 mg vs. 5 mg 52-week | |  |  |
| Aronson 2018 | 1.07 [0.73, 1.58] | p = 0.73 | p = 0.31; I² = 14% |
| Dagogo-Jack 2018 | 0.87 [0.62, 1.22] | p = 0.42 | p = 0.56; I² = 0% |
| Hollander 2018 | 1.00 [0.54, 1.88] | p = 0.99 | p = 0.13; I² = 51% |
| Pratley 2018 | 0.99 [0.54, 1.81] | p = 0.98 | p = 0.13; I² = 51% |
| 15 mg vs. 5 mg 104-week | |  |  |
| Gallos 2019 | 1.00 [0.65, 1.52] | p = 0.99 | NA |
| Hollander 2019 | 2.47 [1.16, 5.23] | p = 0.02 | NA |

RR: Risk Ratio; CI: Confidence Interval; NA: Not Available.
